# Supplementary material for: Photochemically induced cyclic morphological dynamics via degradation of autonomously produced, self-assembled polymer vesicles
Source: Commun Chem. 2021 Feb 26;4:25. doi: 10.1038/s42004-021-00464-8 (PMC9814595; doi:10.1038/s42004-021-00464-8)
Supplement: Supplementary file 2 — Supplementary Information [file 42004_2021_464_MOESM2_ESM.pdf]

Photochemically Induced Cyclic Morphological Dynamics via Degradation of Autonomously Produced Self-Assembled Polymer Vesicles

Supplementary Information File

Chenyu Lin,[1] Sai Krishna Katla,[1] Juan Pérez-Mercader[*1,2]

[1]Department of Earth and Planetary Sciences, Harvard University, Cambridge, Massachusetts 02138-1204, United States

[2]The Santa Fe Institute, Santa Fe, New Mexico 87501, United States

Supplementary Figures

Fig. S 1: Characterization of PEG-*b*-PHPMA block copolymers.

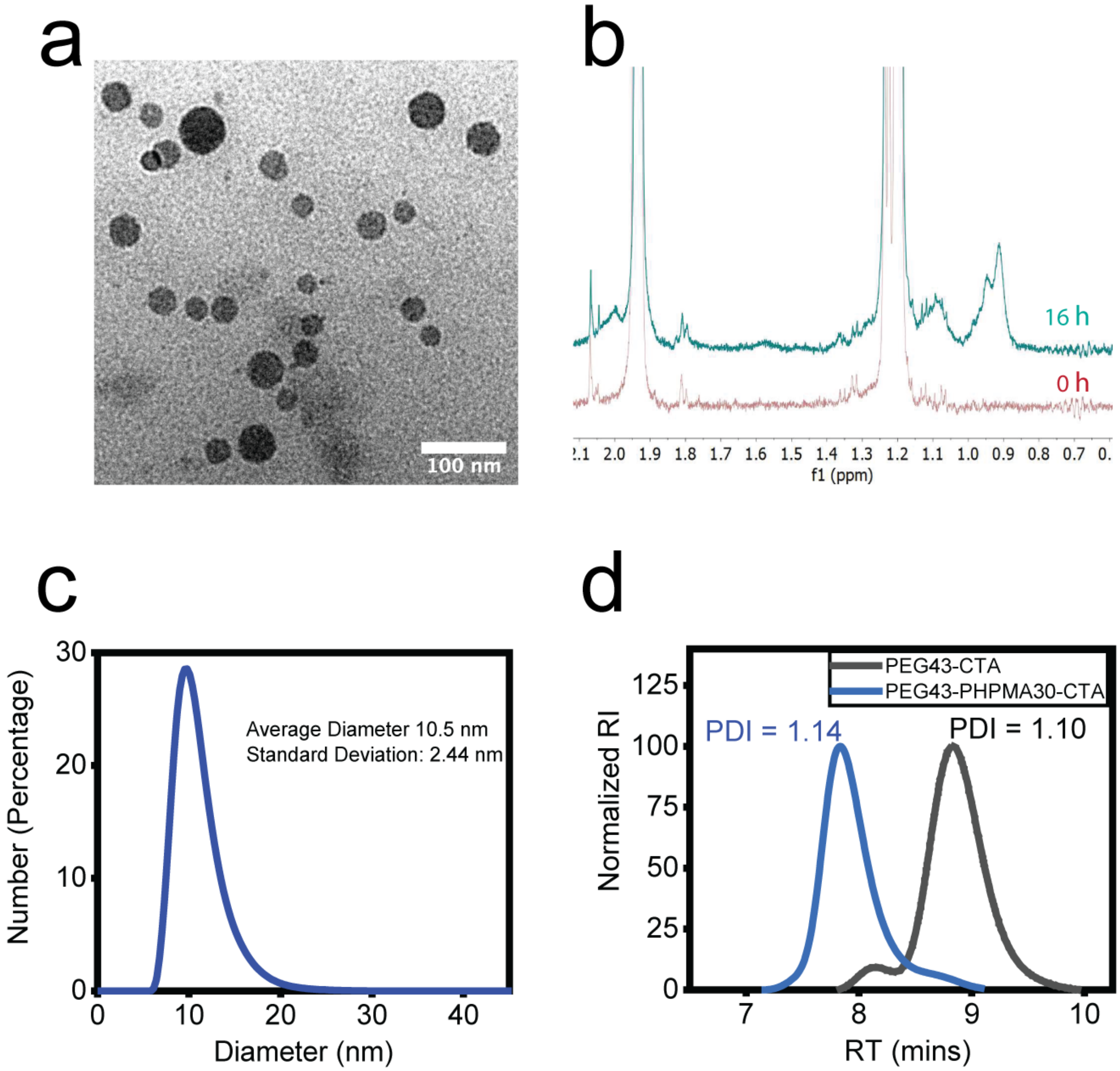

Self-assembled nano-objects formed from the PET-RAFT PISA reaction: **a** TEM image of micelles (scale bar: 100 nm), **b** a portion of ^{1}H-NMR spectrum for block copolymers (black line: before polymerization; green line: after polymerization; the signature peaks located between 0.75 and 1.2 ppm represent the methyl group protons on the PHPMA blocks, Degree of polymerization: 20) **c** DLS confirms the hydrodynamic diameter to be 10.5 nm with standard deviation of 2.44 nm, **d** GPC shows the PDI (1.10) of PEG blocks before polymerization (black curve) and the PDI (1.14) of PEG-b-PHPMA copolymers after polymerization (blue curve).

Fig. S 2: Transmission electron microscopy for giant vesicles.

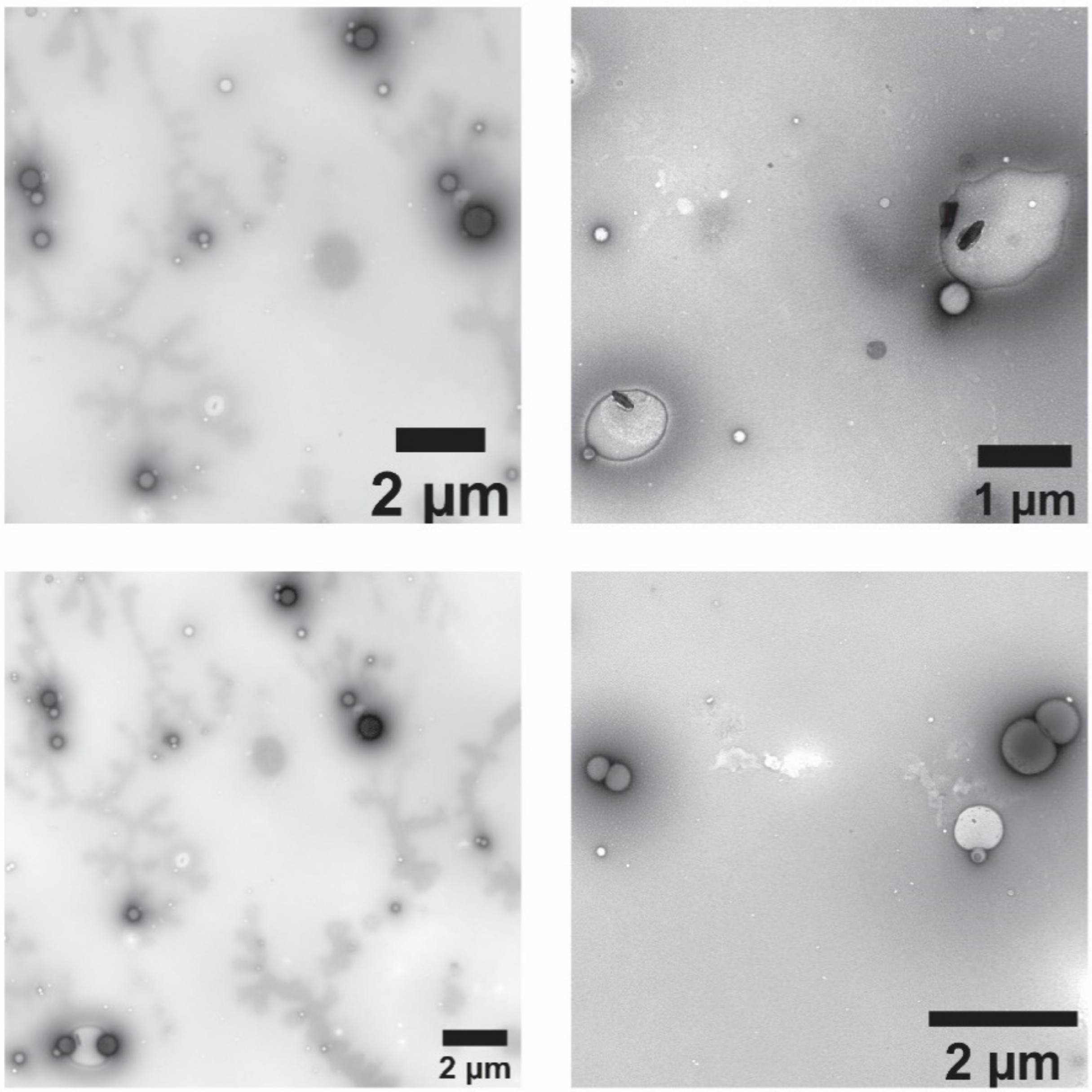

TEM images of the giant vesicles from oxygen-rich PISA specimens after irradiation from microscope.

Fig. S 3: Vesicle population growth curve.

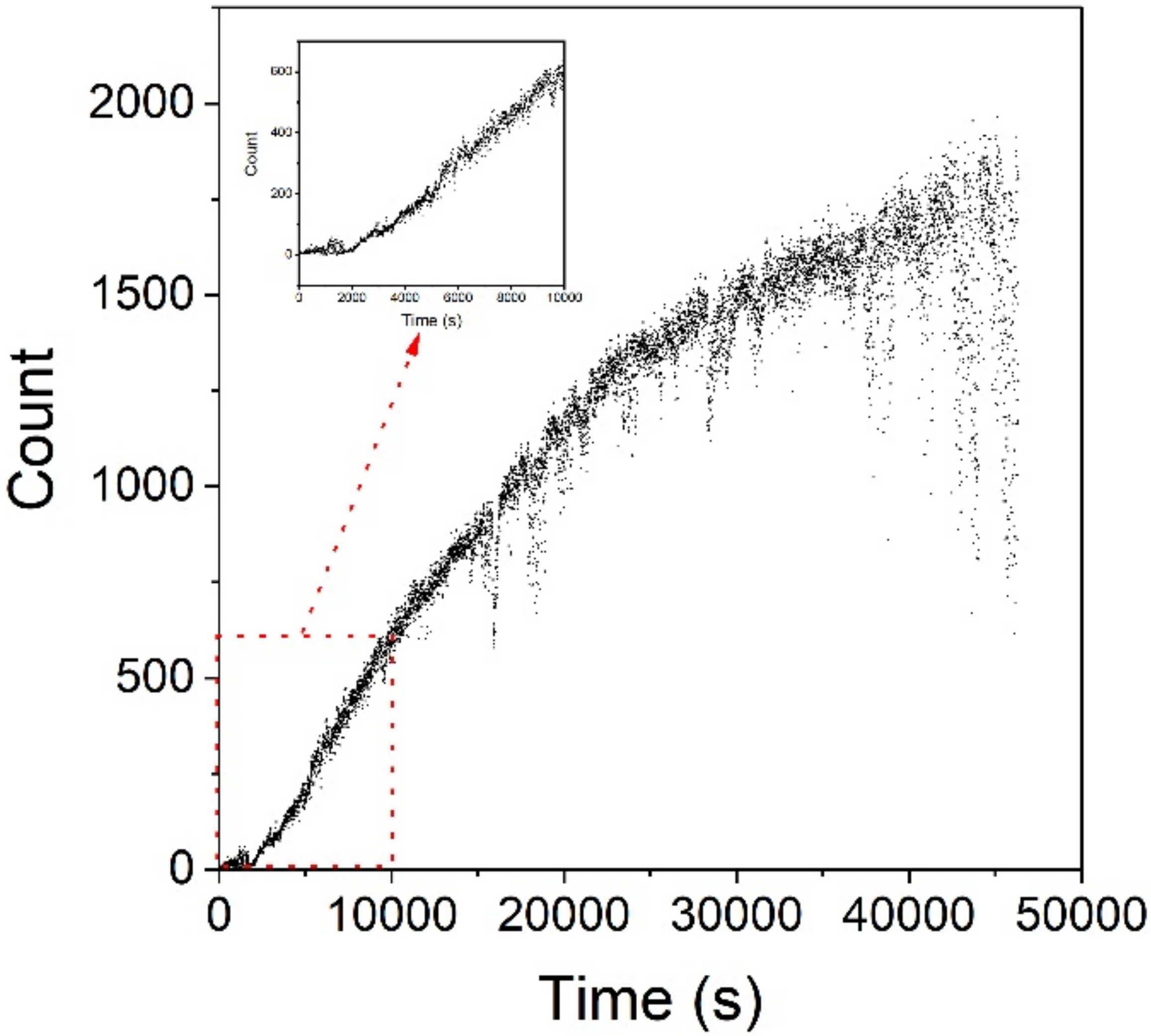

Growth curve of giant vesicle population in an oxygen rich specimen under blue light irradiation from microscope. The count of vesicles in the individual time-lapse images was obtained using ImageJ software.

Fig. S 4: Fluorescence images for temperature effects on phoenix dynamics.

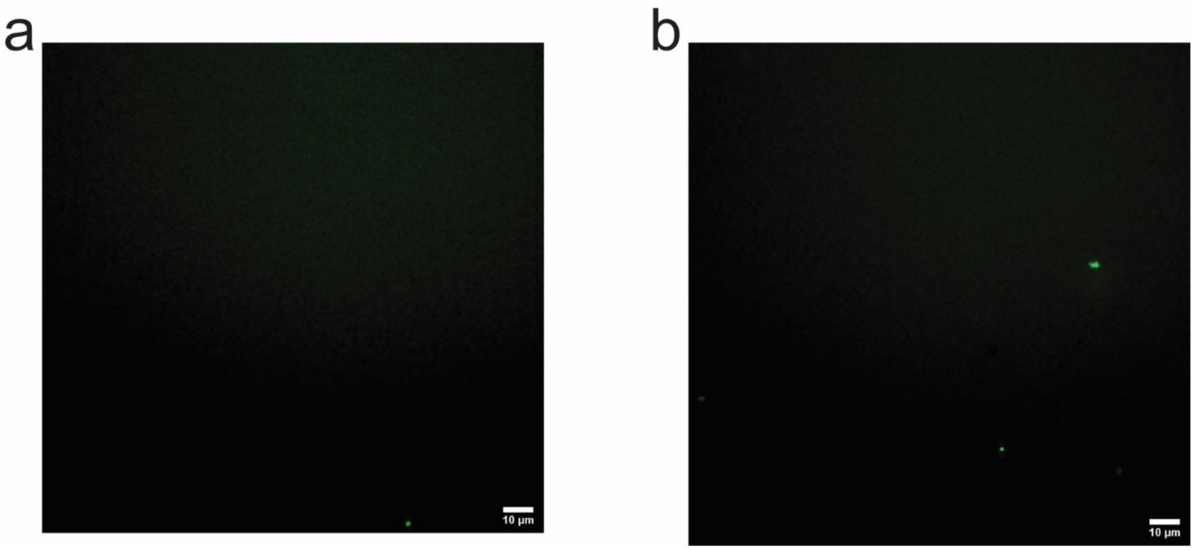

Fluorescence images of air-bubbled PISA specimens which were incubated at 40 °C: **a** After 30 min incubation, **b** After 16-h incubation. The PISA specimens were kept in dark during incubation period.

Fig. S 5: UV-VIS spectroscopy for photo-induced degradation of m-RAFT aqueous solution.

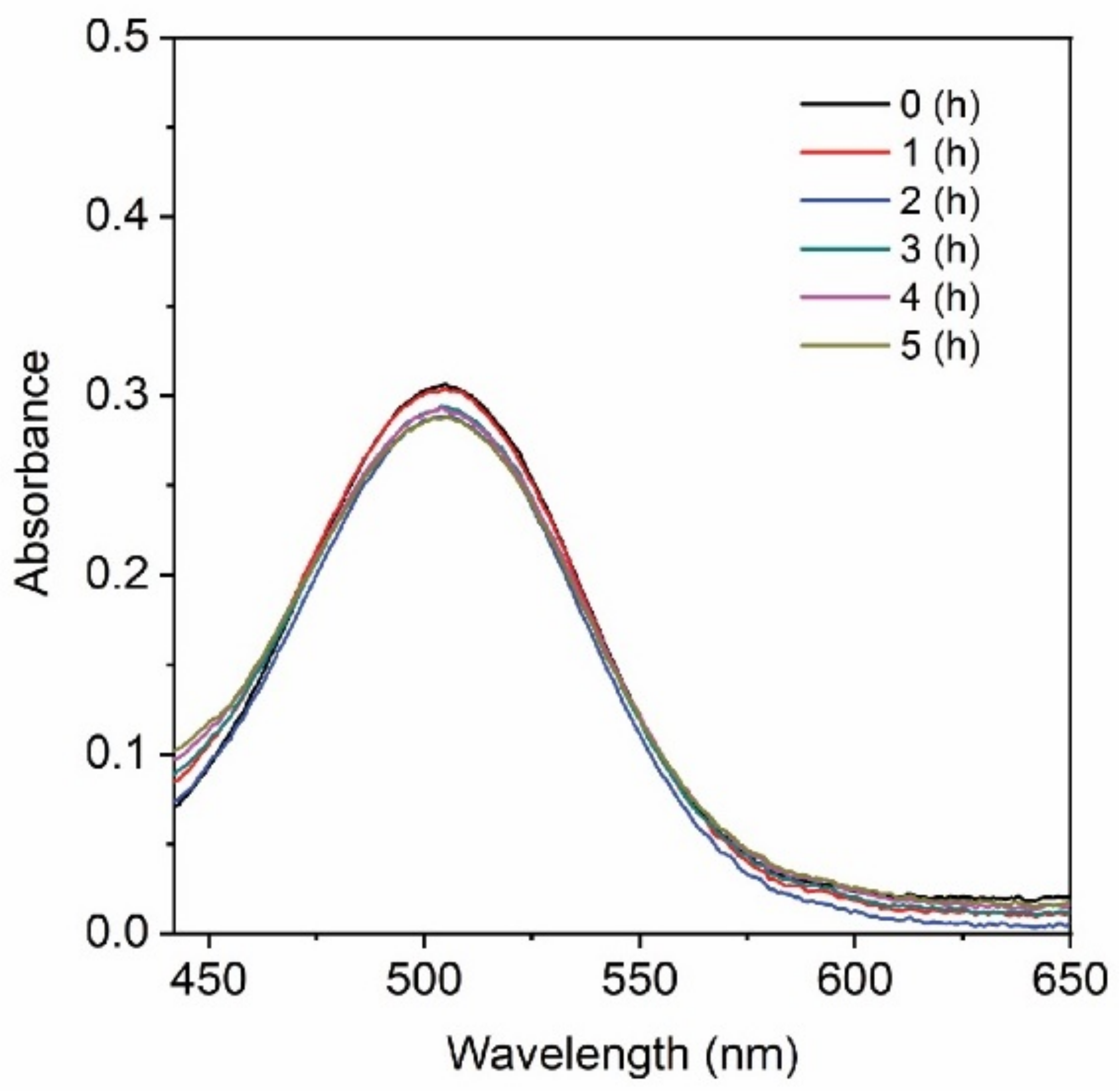

Photo-induced degradation product of m-RAFT aqueous solution prepared in an oxygen-poor environment.

Fig. S 6: Fluorescence images for the effects of in-situ photo-induced degradation.

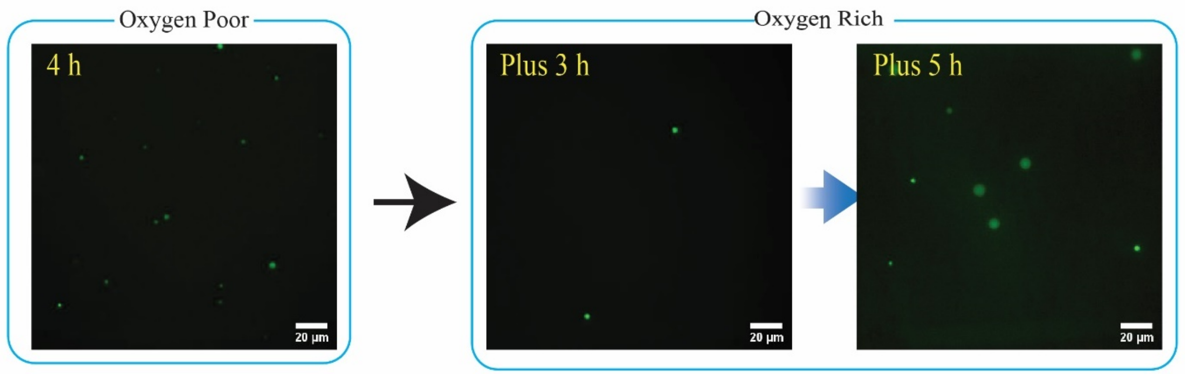

Fluorescence images of oxygen-poor PET-PISA process in reactors. The first 4 h was PET-PISA process using styrene as the monomer in an oxygen poor environment. Afterward, the reactor was exposed to stronger power of blue light with introduction of air and rhodamine 6G. Unlike the use of HPMA, the polymer objects with PS as the hydrophobic cores tend to remain in droplet forms and very few objects show multi-compartmentalization during additional irradiation time.

Fig. S 7: Trace of object diameters and trajectories over time.

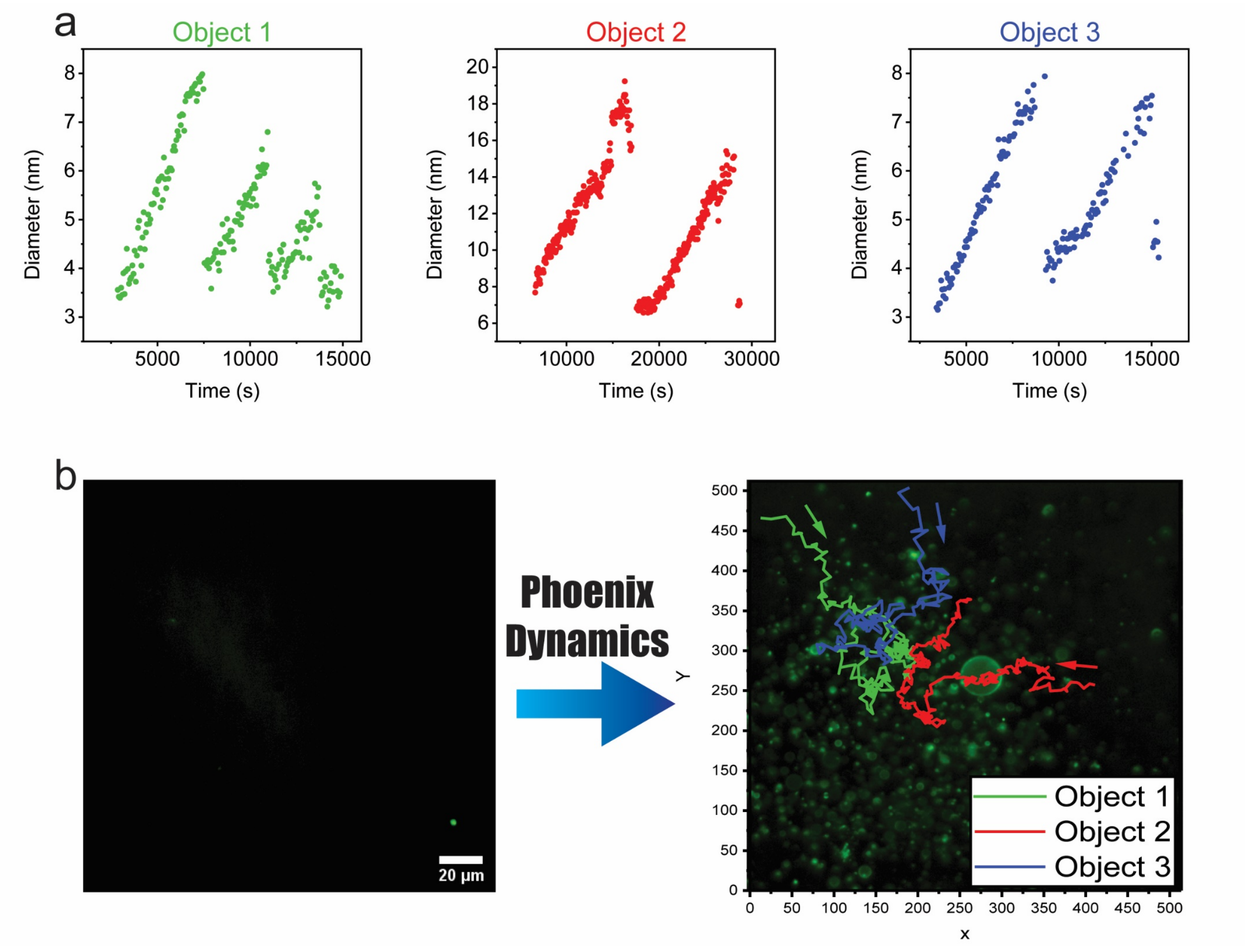

The analysis of size of PISA objects during the Phoenix dynamics **a** Plots of the diameter variation with time for three objects from an oxygen-rich PISA specimen when exposed to irradiation under microscope. **b** Traces of the moving objects shown in **a**. Newborn objects tend to appear in the area where these objects undergoing Phoenix dynamics move.
