# Supplementary material for: Photochemically induced cyclic morphological dynamics via degradation of autonomously produced, self-assembled polymer vesicles
Source: Commun Chem. 2021 Feb 26;4:25. doi: 10.1038/s42004-021-00464-8 (PMC9814595; doi:10.1038/s42004-021-00464-8)
Supplement: Supplementary file 3 — Description of Additional Supplementary Files [file 42004_2021_464_MOESM3_ESM.pdf]

Description of Additional Supplementary Files

File Name: Supplementary Movie 1
Description: A video clip for an oxygen-poor PISA specimen undergoing 16-h blue light irradiation from Zeiss microscope. Pseudo color: green.

File Name: Supplementary Movie 2
Description: A video clip for an oxygen-rich PISA specimen undergoing 16-h blue light irradiation from Zeiss microscope. Pseudo color: green.
